# Screening Mild and Major Neurocognitive Disorders in Parkinson's Disease

**DOI:** 10.1155/2015/983606

**Published:** 2015-05-19

**Authors:** Tivadar Lucza, Kázmér Karádi, János Kállai, Rita Weintraut, József Janszky, Attila Makkos, Sámuel Komoly, Norbert Kovács

**Affiliations:** ^1^Institute of Behavioral Sciences, Faculty of Medicine, University of Pécs, Szigeti Utca 12, Pécs 7624, Hungary; ^2^Department of Neurology, Faculty of Medicine, University of Pécs, Rét Utca 2, Pécs 7623, Hungary; ^3^MTA-PTE Clinical Neuroimaging MR Research Group, Pécs, Rét Utca 23, Pécs 7623, Hungary

## Abstract

*Introduction*. Among the nonmotor features of Parkinson's disease (PD), cognitive impairment is one of the most troublesome problems. New diagnostic criteria for mild and major neurocognitive disorder (NCD) in PD were established by Diagnostic and Statistical Manual of Mental Disorders 5th edition (DSM-5). The aim of our study was to establish the diagnostic accuracy of widely used screening tests for NCD in PD. *Methods*. Within the scope of our study we evaluated the sensitivity and specificity of different neuropsychological tests (Addenbrooke's Cognitive Examination (ACE), Mattis Dementia Rating Scale (MDRS), Mini Mental State Examination (MMSE), and Montreal Cognitive Assessment (MoCA)) in 370 PD patients without depression. *Results*. MoCA and ACE feature the finest diagnostic accuracy for detecting mild cognitive disorder in PD (DSM-5) at the cut-off scores of 23.5 and 83.5 points, respectively. The diagnostic accuracy of these tests was 0.859 (95% CI: 0.818–0.894, MoCA) and 0.820 (95% CI: 0.774–0.859, ACE). In the detection of major NCD (DSM-5), MoCA and MDRS tests exhibited the best diagnostic accuracy at the cut-off scores of 20.5 and 132.5 points, respectively. The diagnostic accuracy of these tests was 0.863 (95% CI: 0.823–0.897, MoCA) and 0.830 (95% CI: 0.785–0.869, MDRS). *Conclusion*. Our study demonstrated that the MoCA may be the most suitable test for detecting mild and major NCD in PD.

## 1. Introduction

Parkinson's disease (PD) is a neurodegenerative disorder characterized by both motor and nonmotor symptoms including depression, fatigue, and autonomic problems. Among nonmotor features, cognitive impairment has one of the most serious consequences by diminishing the quality of life and requiring an increase in caregiver's burden [1–4]. Based on the findings of long-term follow-up studies, neurocognitive impairment unavoidably evolves by disease progression [5, 6]. According to some recent studies, the prevalence of dementia in PD is approximately 20–40% [7]; furthermore, patients with PD have a sixfold risk to develop dementia compared to healthy controls [8].

Although detection of neurocognitive impairment is of the utmost clinical importance, this task is difficult to accurately perform [9]. One of the problems is the heterogeneity of definitions. Previously, the Movement Disorders Society (MDS) Task Force created definitions for dementia in PD (PDD) [9] and mild cognitive impairment in PD (PD-MCI) [10–12]. Moreover, the PD-MCI diagnosis can also be based on two assessment levels: abbreviated (Level I) and comprehensive (Level II) [10–12].

Although the MDS criteria for PDD and PD-MCI have been highly recognized by movement disorder specialists, there are some countries where the diagnosis of cognitive impairment has to be determined by psychiatrists. Until the publication of the most recent version of Diagnostic and Statistical Manual of Mental Disorders (5th edition, DSM-5) in 2013 [13], the diagnosis of PD-MCI was impossible from psychiatric point of view due to the lack of appropriate definitions. According to DSM-5, minor and major neurocognitive disorders (NCD) in PD may be diagnosed which can be considered as the equivalent versions for PD-MCI and PDD, respectively. Comparing to the previous version (DSM 4th edition text revision, DSM-IVTR), the establishment of mild and major NCD in PD is an important enhancement, because previously only the dementia in PD was defined. Therefore, a less severe level of cognitive impairment could not be coded and diagnosed by DSM-4TR. The newly recognized term of mild NCD due to PD may facilitate research and change clinical practice (e.g., patient selection for deep brain stimulation surgery).

According to DSM-5 mild and major NCD may be diagnosed if evidence of significant or modest cognitive decline from a previous level of performance in one or more cognitive domains can be established, respectively. This evidence must be supported by both the concern of the individual, a knowledgeable informant, or the examining clinician noting a significant decline in cognitive function, and a substantial impairment in cognitive performance, preferably documented by standardized neuropsychological testing or, in its absence, another quantified clinical assessment. In the case of major NCD the cognitive deficits interfere with independence in everyday activities, whereas in minor NCD the cognitive deficits do not interfere with capacity for independence in everyday activities, but greater effort, compensatory strategies, or accommodation may be required.

The problem with the establishment of diagnosis is the lack of appropriate clinical screening testing. An ideal screening test should be brief, fast, and appropriately sensitive and specific for detecting subjects possessing characteristics of cognitive impairment [14].

Detection of minor and major NCD in Parkinson's disease is an important task, because cognitive decline is a frequent and important exclusion criteria for deep brain stimulator implantation [15]. Therefore, the necessity of proper screening for cognitive impairment in PD is highly encouraged in clinical practice.

Currently, Mini Mental State Examination (MMSE) is the most commonly used tool for screening cognitive abilities in Hungary [16, 17]. Although it can evaluate orientation, memory, visual abilities, attention and calculation, language, writing, reading, and constructive capabilities, it is not sensitive enough for identifying frontal and executive deficits, and visuospatial dysfunctions. Moreover, it has a limited poor sensitivity for detecting dementia in early stages [18, 19] and it is also unable to differentiate between major types of dementia if applied alone [20]. Although MMSE has been translated and validated into many languages and used in many countries [21], it remains unsuitable for judging eligibility for deep brain stimulation of the subthalamic nuclei (STN DBS) [14, 22]. Best cut-off value for MMSE is 26 points with the sensitivity of 79.9% and specificity of 74.0% for detecting PDD [14], but for PD-MCI it remains unsuitable for screening [23].

Therefore, other dementia screening tests are needed in clinical practice. Although Addenbrooke's Cognitive Examination (ACE) is able to detect early stages of dementia and differentiate some subtypes, its applicability is limited in PD by the lack of widely applicable normative data [14]. ACE also evaluates the major domains of PDD such as orientation, attention and mental flexibility, episodic and semantic memory, verbal fluency, phonemic and semantic category, aphasia tasks, and visuospatial and constructional ability. However, ACE was initially developed by Mathuranath et al. for differentiating between Alzheimer's disease (AD) and frontotemporal dementia (FTD) [24]. The maximum score on ACE is 100 points. ACE was translated into many languages including Hungarian, but it has mainly been tested in AD and not in PD. ACE was validated in PD in some countries [25]. It has limited (<80%) specificity for detecting PDD (the best cut-off score identifying PDD was 80 points, sensitivity = 74.0%, specificity = 78.1%, positive predictive value = 67.42%, negative predictive value = 83.42%) [14]. Therefore, both the original and the revised version of ACE (ACE-R) must be used cautiously as a screening tool for PD-MCI, with results largely influenced by its fluency subdomain score and patient education levels [26, 27].

Mattis Dementia Rating Scale (MDRS) is also a widely used screening instrument for evaluating dementia. It can measure the domains of attention, initiation and perseveration, construction, conceptualization, and memory. MDRS seems to be sensitive for mediotemporal and frontal pathology [28, 29]. Respectfully, the authors generally agree it is one of the most frequently used screening tools for judging cognitive impairment in European DBS centers [30, 31]. Moreover, it is a recommended scale for PD-MCI and PD-D according to MDS criteria at Level I [11, 32]. Its maximum obtainable score is 144 points, whereas the cut-off scores for dementia in French and Spanish PD population was 130 and 123 points, respectively [30, 33]. For the Hungarian version, MDRS had a good sensitivity and specificity for detecting PDD (sensitivity = 89.8%, specificity = 98.3%) using the cut-off score of 125 point [14]. Because the administration of MDRS requires a lengthy period (approximately 30–45 minutes) in clinical setting, MDRS is not an appropriate tool to identify dementia in PD in all occasions.

Montreal Cognitive Assessment (MoCA) was developed as a brief, 10-minute bedside assessment tool for detection of mild cognitive impairment and dementia in AD. It measures 7 domains of cognitive functioning, including visuospatial/executive functions, naming, memory, attention, language, abstraction, and orientation [34]. Comparing to MMSE, MoCA is a more sensitive tool for testing executive, visuospatial functions and attention, which areas are most often impaired in PD. MoCA also has high interrater, test-retest reliability [35], and good discriminant validity for assessing dementia in PD [36, 37]. Further studies demonstrate that MoCA is able to assess broader cognitive domains, and it has higher sensitivity for detecting mild cognitive impairment and dementia in PD [38, 39]. Larner demonstrated better sensitivity and specificity of ACE-R and MoCA over MMSE by comparison [40]. MoCA has been translated and validated into several languages [41–44] and has several alternative forms to overcome the potential practice effects [45, 46].

With reference to the authors' awareness none of the above mentioned screening tools were validated against the diagnostic criteria of DSM-5. Therefore, the aim of our study was to establish the diagnostic accuracy of Montreal Cognitive Assessment (version 7.2) as compared to other widely used screening tests for detecting mild and major neurocognitive disorder in a large sample of Hungarian PD patients.

## 2. Methods

### 2.1. Participants

Four hundred and seventy-two consecutive PD patients treated at Department of Neurology, University of Pécs, were recruited for this study. Each patient fulfilled the clinical diagnostic criteria for idiopathic PD [47]. All of the subjects provided a written informed consent according to the approval of the Regional Ethical Board of University of Pécs.

History of cerebrovascular disease, alcoholism, or other conditions known to impair mental status besides PD served as exclusion criteria for participation. Each patient had a routine brain MRI (or brain CT if MRI was contraindicated). Patients with focal abnormalities on neuroimaging studies, abnormalities in thyroid hormone levels, or noncompensated systemic diseases (i.e., diabetes, hypertension, heart failure) were also excluded.

### 2.2. Patient Evaluation

Patients were evaluated using Hungarian versions of Lille Apathy Scale (LARS) [48], Montgomery-Asberg Depression Rating Scale (MADRS) [49], MMSE [14, 16], ACE [24, 50], MDRS [28], and MoCA [44]. Severity of the Parkinsonian symptoms was assessed by the Hoehn & Yahr (HYS) [51] and the Hungarian validated version of Movement Disorders Society Unified Parkinson's Disease Rating Scales (MDS-UPDRS) [52, 53]. The administering of the applied tests was alternated one from the other to diminish the potential of sequential effect.

Patients suffering from depression were excluded from clinical investigation (score > 18 on MADRS and/or fulfilling the criteria of DSM-5 for depression) to minimize the impact of affective syndromes on cognitive performance. Subsequently, the nondepressed PD patients were divided into three groups based on the fulfillment of the clinical diagnostic criteria for minor and major NCD in PD: patients with major neurocognitive disorder (major NCD group), patients with minor neurocognitive disorder (minor NCD group), and patients without either minor or major neurocognitive disorder (normal PD group) [13]. To increase reliability, a single experienced investigator (NK) categorized each patient into normal, minor NCD, and major NCD groups.

### 2.3. Data Analysis

Statistical analyses were performed by The R Project for Statistical Computing (Windows version 3.1.2) [54]. Because most data did not follow the normal distribution, nonparametric tests (Kruskal-Wallis tests) were applied. Since HYS represents an ordinal scale, Chi-square test was applied for analyses involving HYS. The level of significance was set at 0.05.

To measure diagnostic accuracy for neurocognitive batteries, Receiver Operating Characteristic (ROC) curve analysis was obtained [55] to measure sensitivity, specificity, positive likelihood ratio, negative likelihood ratio, positive predictive value, negative predictive value, and diagnostic accuracy.

Because the ideal cut-off is one which selects an immense amount of disease (high sensitivity) but has very few false positives (high specificity), we chose the best cut-off point for balancing the sensitivity and specificity by identifying the point on the curve closest to the (0, 1) point.

## 3. Results

One hundred and two patients expressed a coexistent depression; therefore, they were excluded from further analyses. Therefore, the data of 370 patients were analyzed subsequently. Out of the 370 evaluated subjects, 257 had normal cognitive profile, 60 had mild neurocognitive disorder, and 53 had major neurocognitive disorder in PD according to DSM-5 classification. The comparison of the demographic and clinical characteristics between normal, mild NC, and major NC groups is presented in Table 1.

Fulfilling our expectations, all the examined dementia screening scales (MDRS, MoCA, MMSE, and ACE) demonstrated significant differences between the normal, mild NC, and major NC groups (Table 1).

**Figure 1:**
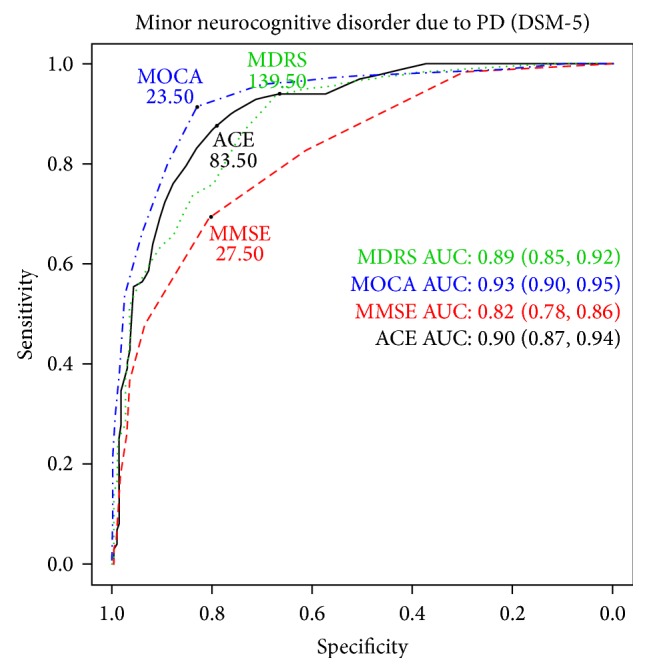
Receiver Operating Characteristic analysis of Addenbrooke's Cognitive Examination (ACE), Mattis Dementia Rating Scale (MDRS), Mini Mental State Examination (MMSE), and Montreal Cognitive Assessment (MoCA) for detecting mild neurocognitive disorder due to Parkinson's disease (PD) in accordance with the Diagnostic and Statistical Manual of Mental Disorders 5th edition criteria. Area under the curve (AUC) values are also represented with the 95% confidence interval values.

Based on ROC analysis, MoCA and ACE had the best diagnostic accuracy for detecting mild NCD in PD at the cut-off scores of 23.5 and 83.5 points, respectively (Table 2, Figure 1). The diagnostic accuracy of these tests was 0.859 (95% confidence interval—CI: 0.818–0.894, MoCA) and 0.820 (95% CI: 0.774–0.859, ACE) meaning 85.9% and 82.0% of true positive and true negative cases are identified. The other variables describing the diagnostic accuracy (specificity, sensitivity, positive and negative predictive values, and positive and negative likelihood ratios) are presented in Table 2 with their 95% CI values. Area under the curve (AUC) values are demonstrated in Figure 1.

**Figure 2:**
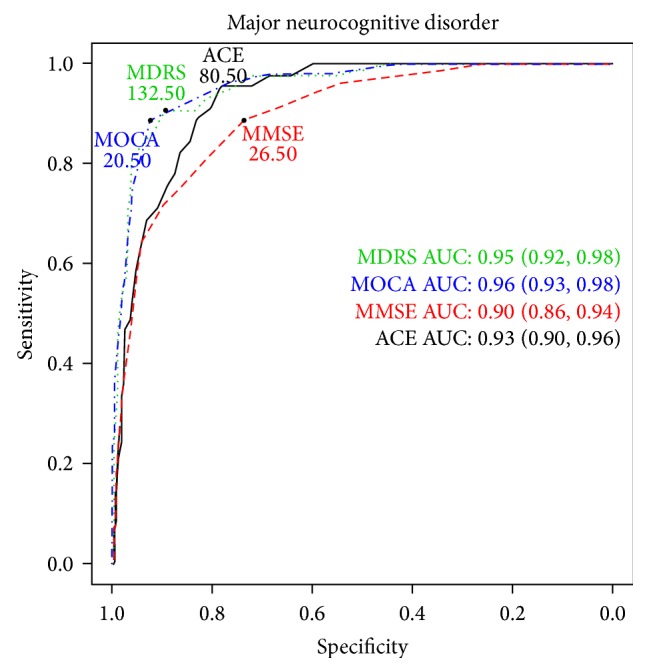
Receiver Operating Characteristic analysis of Addenbrooke's Cognitive Examination (ACE), Mattis Dementia Rating Scale (MDRS), Mini Mental State Examination (MMSE), and Montreal Cognitive Assessment (MoCA) for detecting major neurocognitive disorder due to Parkinson's disease (PD) in accordance with the Diagnostic and Statistical Manual of Mental Disorders 5th edition criteria. Area under the curve (AUC) values are also represented with the 95% confidence interval values.

For detecting major NCD, MoCA and MDRS tests exhibited the highest diagnostic accuracy at the cut-off scores of 20.5 and 132.5 points, respectively (Table 3 and Figure 2). The diagnostic accuracy of these tests was 0.863 (95% CI: 0.823–0.897, MoCA) and 0.830 (95% CI: 0.785–0.869, MDRS) meaning 86.3% and 83.0% of true positive and true negative cases are identified. The other variables describing the diagnostic accuracy (specificity, sensitivity, positive and negative predictive values, and positive and negative likelihood ratios) are presented in Table 3 with their 95% CI values. AUC values are demonstrated in Figure 2.

## 4. Discussion

Screening for NCD in Parkinson's disease is an important clinical necessity for establishing diagnosis and initiating effective and proper treatment. In differentiating between normal cognition from impaired cognitive abilities an easily applicable, reproducible, and validated test battery with high diagnostic accuracy is needed. The aim of our study was to establish the diagnostic accuracy of widely used screening tests for detecting mild and major neurocognitive disorder in PD.

Although some major demographic and PD-related properties (e.g., education, sex, disease duration, and anti-Parkinson medication expressed in levodopa equivalent dosage and severity of depressive symptom measured by MADRS) were comparable, patients with either minor or major neurocognitive disorder were older and had higher age at disease onset and more severe Parkinsonian symptoms (MDS-UPDRS and HYS). This is not a surprising factor, because age and age of disease onset are significant factors for developing major neurocognitive disorder, and the presence of cognitive impairment is associated with more severe gait and postural instability resulting in higher HYS and MDS-UPDRS Part 3 (Motor Examination) values [56].

To the best of the author's knowledge, this study is the first validating the most popular screening tests against the recently developed mild and major NCD due to PD established by the DSM-5. Based on our results, MoCA had better diagnostic accuracy for detecting mild NCD in PD based on the DSM-5 criteria. Scores <139.5 on MDRS or <83.5 on ACE or <23.5 on MoCA can suggest the presence of mild NCD in PD (DSM-5). Recent studies revealed similar cut-off values for MDRS. Villeneuve et al. suggested a normality cut-off of 138 on the MDRS having the sensitivity of 72% and specificity of 86% with a correct classification of 80% for detecting PD-MCI (MDS Task Force criteria) [57]. One of the limitations of their study was, however, the relatively low number of PD patients involved (*n* = 40). Pirogovsky et al. published a different study on MDRS having the sample of 30 patients diagnosed with PD-MCI based on Level II MDS criteria and 68 PD patients with normal cognition. They suggested that a total score of ≤139 for screening purposes yielded a better balance between sensitivity (77%) and specificity (65%) [58].

Previously our team also validated MDRS against the PDD criteria (MDS Task Force criteria) [14]. In this former study we suggested the usage of 125 points as a cut-off score for diagnosing PDD. However, in the present study we recommended the cut-off of 132.5 points to diagnose major NCD in PD. This apparent difference might be due to the larger sample size (370 versus 73), the difference between the applied diagnostic criteria (major NCD according to DSM-5 versus PDD established by MDS Task Force), and the discrepancies between the mean educational levels (11.9 ± 4.4 versus 13.0 ± 3.8 years).

Although MMSE is still one of the most frequently used screening tests, it is generally considered unsuitable for reliable NCD identification in PD [22]. Despite the MoCA and MMSE scores which can be converted [59], MoCA, MDRS, and ACE tests appear to be generally superior for screening NCD in PD [14, 23, 37, 57]. PD subjects with normal range MMSE but abnormal MoCA scores had evidence of caudate nucleus dopaminergic denervation and mild cognitive changes, predominantly in executive function [60]. PD patients with borderline cognitive impairment have impairments in their decisional capacity. The MoCA may be useful to identify the patients at risk of impaired capacity [61].

The Parkinson Study Group Cognitive/Psychiatric Working Group recommended MoCA as a minimum cognitive screening measure in clinical trials of PD where cognitive performance is not the primary outcome measure [22]. The commonly recommended cut-off screening score for dementia of 26 on the MoCA is too high for PD patients and most studies suggest the utilization 23-24 points for cut-off [62].

However, the application of MoCA in PD-MCI remains controversial. A recent study [63] demonstrated that MoCA is suitable for screening large population for Parkinson's disease dementia (PDD, according to MDS Task Force criteria [9]). On the contrary, other studies showed that when decline from estimated premorbid levels was considered evidence of cognitive impairment (Level 2 criteria for PD-MCI), both MoCA and MMSE had poor diagnostic accuracy for PD-MCI (65.3%) [60]. At the lowest cut-off levels that provided at least 80% sensitivity, specificity was low (44%) for the Montreal Cognitive Assessment [23]. Therefore the authors concluded that MoCA may be able to preferentially detect executive dysfunction compared to the MMSE, but the MoCA has limited diagnostic accuracy for PD-MCI and should not solely be used to substantiate this diagnosis.

The MoCA may be more sensitive than the MMSE in detecting early baseline and longitudinal cognitive impairment in PD. Based on the analysis of 95 patients, a MoCA score of ≤26 provided a sensitivity of 93.1% for the diagnosis of PD-MCI [64]. In the longitudinal cohort, baseline MoCA was useful in predicting cognitive decline over 2 years [64]. A baseline MoCA ≤26 was highly predictive of progressive cognitive decline (HR 3.47, 95% CI: 2.38–5.07; *p* < 0.01) over 2 years. This finding was also confirmed by another study [65]. The longitudinal data from 155 patients with PD over 18 months showed significant reductions in MoCA scores, but not in MMSE scores, with 21.3% of patients moving from normal cognition to MCI and 4.5% moving from MCI to dementia.

## 5. Conclusions

DSM-5 criteria for diagnosing mild and major NCD in PD are clinically feasible. Although most popular screening tests including MoCA, MDRS, and ACE are proven useful for screening patients, in the risk population the accurate diagnosis should be based on appropriate neuropsychological evaluation.

## Figures and Tables

**Table 1:** Demographic, Parkinson's disease- (PD-) related and neurocognitive data of the study population.

	Normal cognition (*n* = 257)					Mild neurocognitive disorder due to PD (*n* = 60)					Major neurocognitive disorder due to PD (*n* = 53)					*p* value
	Median	Percentile 25	Percentile 75	Mean	SD	Median	Percentile 25	Percentile 75	Mean	SD	Median	Percentile 25	Percentile 75	Mean	SD	
Age (years)	65	59	70	63.73	10.13	71	65	76	70.35	7.33	74	70	79	74.28	7.03	0.000
Age at onset (years)	57	49	65	56.00	11.69	64	54	68	61.97	10.27	67	56	75	65.25	12.47	0.000
Education (years)	12	11	16	13.04	3.08	12	11	15	12.28	3.00	12	11	15	12.00	2.97	0.124
Sex (male/female)	201 M/56 F					47 M/13 F					42 M/11 F					0.986
Disease duration (years)	6	2	11	7.58	6.74	7	3	12	8.38	6.68	7	3	15	9.08	7.56	0.328

LED (mg)	400	14	885	559.30	544.13	330	14	765	506.80	614.24	334	14	780	490.94	558.59	0.642
MDS-UPDRS Part 1	12	9	17	12.63	5.96	13	10	18	14.13	6.88	13	11	20	13.44	6.64	0.077
MDS-UPDRS Part 2	13	7	19	13.93	8.65	13	9	19	13.35	7.83	16	9	22	15.92	7.83	0.137
MDS-UPDRS Part 3	37	28	46	38.22	14.73	40	34	51	42.07	14.56	50	41	58	48.72	13.55	0.000
MDS-UPDRS Part 4	4	2	6	4.77	3.50	4	2	6	4.12	3.06	4	2	6	4.57	3.21	0.423
Hoehn & Yahr Stage (1/2/3/4/5)	5/157/67/24/4					0/30/20/9/1					0/15/24/11/3					0.001

MADRS	8	5	13	9.48	5.79	10	5	15	10.08	5.97	10	6	15	10.68	5.48	0.092
LARS	−25	−30	−21	−24.01	7.61	−22	−28	−17	−21.91	7.02	−19	−26	−11	−17.79	10.97	0.000

ACE orientation	10	10	10	9.93	0.48	10	10	10	9.81	0.50	10	9	10	9.16	1.30	0.000
ACE attention	8	8	8	7.92	0.40	8	7	8	7.68	0.59	7	6	8	6.78	1.47	0.000
ACE memory	29	26	32	28.41	4.97	23	19	26	22.26	5.73	19	14	24	18.86	6.44	0.000
ACE verbal fluency	10	9	12	10.01	2.48	8	6	9	7.53	2.22	6	4	7	6.05	2.41	0.000
ACE language	28	27	28	27.53	0.97	27	27	28	27.06	0.94	26	26	28	25.89	2.60	0.000
ACE visuospatial	5	4	5	4.23	1.00	4	3	4	3.38	1.21	2	2	3	2.59	1.19	0.000
ACE total score	89	85	93	88.02	7.58	79	73	82	77.72	6.79	70	63	76	69.35	8.88	0.000

MMSE orientation	10	10	10	9.89	0.75	10	10	10	9.85	0.45	10	8	10	9.08	1.25	0.000
MMSE attention	8	8	8	7.90	0.46	8	8	8	7.66	0.82	7	5	8	6.51	1.67	0.000
MMSE memory	2	1	3	2.07	0.91	2	1	2	1.49	0.84	1	0	2	1.02	0.95	0.000
MMSE language	8	8	8	7.71	0.64	8	7	8	7.53	0.82	7	7	8	7.04	1.14	0.000
MMSE visuospatial	1	1	1	0.93	0.25	1	1	1	0.81	0.39	0	0	1	0.49	0.50	0.000
MMSE total score	29	28	30	28.51	1.98	28	26	29	27.34	1.71	25	22	27	24.13	3.09	0.000

MoCA Executive/visuospatial	4	4	5	4.13	0.86	3	3	4	3.14	1.09	2	2	3	2.49	1.10	0.000
MoCA naming	3	3	3	2.99	0.12	3	3	3	2.90	0.36	3	3	3	2.79	0.41	0.000
MoCA attention	6	5	6	5.45	0.84	5	4	6	4.75	1.04	3	3	4	3.49	1.42	0.000
MoCA language	2	2	3	2.35	0.72	2	1	2	1.63	0.91	1	1	2	1.34	0.81	0.000
MoCA abstraction	2	2	2	1.90	0.31	2	2	2	1.66	0.66	2	1	2	1.57	0.67	0.000
MoCA delayed recall	3	1	4	2.44	1.48	1	0	2	1.14	1.20	0	0	1	0.49	0.93	0.000
MoCA orientation	6	6	6	5.97	0.17	6	6	6	5.92	0.28	5	5	6	5.17	1.01	0.000
MoCA total score	26	24	27	25.70	2.28	22	20	23	21.71	2.18	18	17	20	18.08	3.02	0.000

Mattis attention	36	36	37	36.09	1.02	35	35	36	35.36	1.01	35	34	36	34.25	2.25	0.000
Mattis initiation/perseveration	37	34	37	35.23	2.72	34	31	37	33.36	3.23	29	26	31	28.39	4.16	0.000
Mattis construction	6	6	6	5.98	0.23	6	6	6	5.94	0.32	6	6	6	5.58	1.42	0.030
Mattis conceptualization	39	39	39	38.74	1.42	39	38	39	38.45	1.08	38	37	39	37.97	1.18	0.000
Mattis memory	24	23	25	23.52	1.84	22	20	24	21.38	3.96	19	17	21	18.56	3.80	0.000
Mattis total score	141	138	143	139.56	4.72	136	131	138	134.94	4.62	125	123	128	124.75	7.03	0.000

Mild and major neurocognitive disorder because Parkinson's disease was defined by the Diagnostic and Statistical Manual of Mental Disorders 5th edition criteria. *p* values are calculated by Kruskal-Wallis test with the exception of Hoehn & Yahr Scale and sex where Chi-square tests were utilized. ACE: Addenbrooke's Cognitive Examination; F: females; LARS: Lille's Apathy Scale; M: males; MADRS: Montgomery-Asberg Depression Rating Scale; MDRS: Mattis Dementia Rating Scale; MDS-UPDRS: Movement Disorders Society's Unified Parkinson's Disease Rating Scale; MMSE: Mini Mental State Examination; MoCA: Montreal Cognitive Assessment.

**Table 2:** Diagnostic accuracy of screening tests for detecting mild neurocognitive disorder due to Parkinson's disease.

	Diagnostic accuracy											
	MoCA			ACE			MMSE			MDRS		
	Estimation	Lower 95% CI	Upper 95% CI	Estimation	Lower 95% CI	Upper 95% CI	Estimation	Lower 95% CI	Upper 95% CI	Estimation	Lower 95% CI	Upper 95% CI
Best cut-off score	23.5			83.5			27.5			139.5		
Sensitivity	0.915	0.848	0.958	0.871	0.790	0.930	0.602	0.540	0.714	0.939	0.873	0.977
Specificity	0.831	0.777	0.877	0.797	0.741	0.847	0.706	0.652	0.723	0.670	0.605	0.730
Positive predictive value	0.733	0.653	0.803	0.647	0.561	0.727	0.623	0.534	0.707	0.550	0.472	0.627
Negative predictive value	0.950	0.911	0.976	0.936	0.892	0.965	0.650	0.598	0.793	0.962	0.920	0.986
Diagnostic accuracy	0.859	0.818	0.894	0.820	0.774	0.859	0.671	0.624	0.712	0.751	0.700	0.797
Likelihood ratio of a positive test	5.417	4.047	7.250	4.302	3.305	5.599	2.575	1.804	3.125	2.843	2.349	3.440
Likelihood ratio of a negative test	0.103	0.057	0.187	0.161	0.097	0.269	0.312	0.259	0.484	0.091	0.041	0.198

Mild neurocognitive disorder because Parkinson's disease was defined by the Diagnostic and Statistical Manual of Mental Disorders 5th edition criteria.

ACE: Addenbrooke's Cognitive Examination; CI: confidence interval; MDRS: Mattis Dementia Rating Scale; MMSE: Mini Mental State Examination; MoCA: Montreal Cognitive Assessment.

**Table 3:** Diagnostic accuracy of screening tests for detecting major neurocognitive disorder due to Parkinson's disease.

	Diagnostic accuracy											
	MoCA			ACE			MMSE			MDRS		
	Estimation	Lower 95% CI	Upper 95% CI	Estimation	Lower 95% CI	Upper 95% CI	Estimation	Lower 95% CI	Upper 95% CI	Estimation	Lower 95% CI	Upper 95% CI
Best cut-off score	20.5			80.5			26.5			132.5		
Sensitivity	0.921	0.860	0.962	0.869	0.784	0.927	0.692	0.600	0.774	0.566	0.462	0.665
Specificity	0.801	0.738	0.859	0.790	0.737	0.841	0.806	0.752	0.853	0.943	0.905	0.970
Positive predictive value	0.750	0.674	0.816	0.640	0.550	0.714	0.623	0.534	0.707	0.812	0.699	0.896
Negative predictive value	0.953	0.917	0.980	0.921	0.874	0.945	0.850	0.798	0.893	0.835	0.784	0.878
Diagnostic accuracy	0.863	0.823	0.897	0.814	0.764	0.879	0.770	0.724	0.812	0.830	0.785	0.869
Likelihood ratio of a positive test	5.457	4.081	7.297	4.284	3.292	5.575	3.575	2.704	4.725	10.008	5.742	17.442
Likelihood ratio of a negative test	0.095	0.052	0.172	0.162	0.097	0.269	0.382	0.289	0.504	0.460	0.367	0.578

Major neurocognitive disorder because Parkinson's disease was defined by the Diagnostic and Statistical Manual of Mental Disorders 5th edition criteria.

ACE: Addenbrooke's Cognitive Examination; CI: confidence interval; MDRS: Mattis Dementia Rating Scale; MMSE: Mini Mental State Examination; MoCA: Montreal Cognitive Assessment.

## Abbreviations

ACE: Addenbrooke's Cognitive Examination
ADL: Activities of Daily Living
AUC: Area under curve
CI: Confidence interval
DSM-5: Diagnostic and Statistical Manual of Mental Disorders 5th edition
HYS: Hoehn & Yahr Scale
LARS: Lille's Apathy Scale
MADRS: Montgomery-Asberg Depression Rating Scale
MDRS: Mattis Dementia Rating Scale
MDS: Movement Disorders Society
MDS-UPDRS: Movement Disorders Society's Unified Parkinson's Disease Rating Scale
MMSE: Mini Mental State Examination
MoCA: Montreal Cognitive Assessment
NCD: Neurocognitive disorder
PD: Parkinson's disease
ROC: Receiver Operating Characteristic.

## References

[1] Reid W. G. (1992). The evolution of dementia in idiopathic Parkinson's disease: neuropsychological and clinical evidence in support of subtypes. *International Psychogeriatrics*.

[2] Tröster A. I., Woods S. P., Morgan E. E. (2007). Assessing cognitive change in Parkinson's disease: development of practice effect-corrected reliable change indices. *Archives of Clinical Neuropsychology*.

[3] Kulisevsky J., Pagonabarraga J. (2009). Cognitive impairment in Parkinson's disease: tools for diagnosis and assessment. *Movement Disorders*.

[4] Dujardin K., Duhamel A., Delliaux M., Thomas-Antérion C., Destée A., Defebvre L. (2010). Cognitive complaints in Parkinson's disease: its relationship with objective cognitive decline. *Journal of Neurology*.

[5] Reid W. G. J., Hely M. A., Morris J. G. L., Loy C., Halliday G. M. (2011). Dementia in Parkinson's disease: a 20-year neuropsychological study (Sydney multicentre study). *Journal of Neurology, Neurosurgery and Psychiatry*.

[6] Hely M. A., Reid W. G. J., Adena M. A., Halliday G. M., Morris J. G. L. (2008). The Sydney multicenter study of Parkinson's disease: the inevitability of dementia at 20 years. *Movement Disorders*.

[7] Marder K. (2010). Cognitive impairment and dementia in Parkinson's disease. *Movement Disorders*.

[8] Aarsland D., Andersen K., Larsen J. P., Lolk A., Kragh-Sørensen P. (2003). Prevalence and characteristics of dementia in Parkinson disease: an 8-year prospective study. *Archives of Neurology*.

[9] Goetz C. G., Emre M., Dubois B. (2008). Parkinson's disease dementia: definitions, guidelines, and research perspectives in diagnosis. *Annals of Neurology*.

[10] Geurtsen G. J., Hoogland J., Goldman J. G. (2014). Parkinson's disease mild cognitive impairment: application and validation of the criteria. *Journal of Parkinson's Disease*.

[11] Litvan I., Goldman J. G., Tröster A. I. (2012). Diagnostic criteria for mild cognitive impairment in Parkinson's disease: movement disorder society task force guidelines. *Movement Disorders*.

[12] Litvan I., Aarsland D., Adler C. H. (2011). MDS task force on mild cognitive impairment in Parkinson's disease: critical review of PD-MCI. *Movement Disorders*.

[13] American Psychiatric Association (2013). *Diagnostic and Statistical Manual of Mental Disorders*.

[14] Kaszás B., Kovács N., Balás I. (2012). Sensitivity and specificity of Addenbrooke's Cognitive Examination, Mattis Dementia Rating Scale, Frontal Assessment Battery and Mini Mental State Examination for diagnosing dementia in Parkinson's disease. *Parkinsonism and Related Disorders*.

[15] Fehér G., Balás I., Komoly S. (2010). Analysis of antiparkinsonian drug reduction after bilateral subthalamic deep brain stimulation. *Ideggyogyaszati Szemle*.

[16] Égerházi A. (2008). The early diagnosis and differential diagnosis of Alzheimer's disease with clinical methods. *Orvosi Hetilap*.

[17] Folstein M. F., Folstein S. E., McHugh P. R. (1975). ‘Mini-mental state’: a practical method for grading the cognitive state of patients for the clinician. *Journal of Psychiatric Research*.

[18] Tombaugh T. N., McIntyre N. J. (1992). The mini-mental state examination: a comprehensive review. *Journal of the American Geriatrics Society*.

[19] Feher E. P., Mahurin R. K., Doody R. S., Cooke N., Sims J., Pirozzolo F. J. (1992). Establishing the limits of the mini-mental state: examination of ‘subtests’. *Archives of Neurology*.

[20] Santacruz K. S., Swagerty D. (2001). Early diagnosis of dementia. *The American Family Physician*.

[21] Magloczky E., Janka Z. (1988). Assessment of dementia in social welfare homes: applicability of the mini-mental state test. *Szociális Gondozó*.

[22] Chou K. L., Amick M. M., Brandt J. (2010). A recommended scale for cognitive screening in clinical trials of Parkinson's disease. *Movement Disorders*.

[23] Marras C., Armstrong M. J., Meaney C. A. (2013). Measuring mild cognitive impairment in patients with Parkinson's disease. *Movement Disorders*.

[24] Mathuranath P. S., Nestor P. J., Berrios G. E., Rakowicz W., Hodges J. R. (2000). A brief cognitive test battery to differentiate Alzheimer's disease and frontotemporal dementia. *Neurology*.

[25] Reyes M. A., Lloret S. P., Gerscovich E. R., Martin M. E., Leiguarda R., Merello M. (2009). Addenbrooke's Cognitive examination validation in Parkinson's disease. *European Journal of Neurology*.

[26] Komadina N. C., Terpening Z., Huang Y., Halliday G. M., Naismith S. L., Lewis S. J. G. (2011). Utility and limitations of Addenbrooke's cognitive examination-revised for detecting mild cognitive impairment in Parkinson's disease. *Dementia and Geriatric Cognitive Disorders*.

[27] Mccolgan P., Evans J. R., Breen D. P., Mason S. L., Barker R. A., Williams-Gray C. H. (2012). Addenbrooke's Cognitive Examination-Revised for mild cognitive impairment in Parkinson's disease. *Movement Disorders*.

[28] Mattis S., Bellack L., Karusu T. B. (1976). Mental status examination for organic mental syndrome in the elderly patient. *Geriatric Psychiatry*.

[29] Brown G. G., Armstrong Rahill A., Gorell J. M. (1999). Validity of the dementia rating scale in assessing cognitive function in Parkinson's disease. *Journal of Geriatric Psychiatry and Neurology*.

[30] Llebaria G., Pagonabarraga J., Kulisevsky J. (2008). Cut-off score of the Mattis Dementia Rating Scale for screening dementia in Parkinson's disease. *Movement Disorders*.

[31] Deli G., Aschermann Z., Acs P. Bilateral subthalamic stimulation can improve sleep quality in Parkinson's disease.

[32] Dubois B., Burn D., Goetz C. (2007). Diagnostic procedures for Parkinson's disease dementia: recommendations from the movement disorder society task force. *Movement Disorders*.

[33] Di Virgilio G., Leroy A., Cunin P., Mahieux F., Bachoud-Levi A., Fenelon G. (2007). The Mini Mental Parkinson brief cognitive test: comparison with the Mattis dementia rating scale in 289 patients with Parkinson’s disease. *Movement Disorder*.

[34] Nasreddine Z. S., Phillips N. A., Bédirian V. (2005). The Montreal Cognitive Assessment, MoCA: a brief screening tool for mild cognitive impairment. *Journal of the American Geriatrics Society*.

[35] Gill D. J., Freshman A., Blender J. A., Ravina B. (2008). The Montreal Cognitive Assessment as a screening tool for cognitive impairment in Parkinson's disease. *Movement Disorders*.

[36] Hoops S., Nazem S., Siderowf A. D. (2009). Validity of the MoCA and MMSE in the detection of MCI and dementia in Parkinson disease. *Neurology*.

[37] Dalrymple-Alford J. C., MacAskill M. R., Nakas C. T. (2010). The MoCA: well-suited screen for cognitive impairment in Parkinson disease. *Neurology*.

[38] Julayanont P., Brousseau M., Chertkow H., Phillips N., Nasreddine Z. S. (2014). Montreal Cognitive Assessment Memory Index Score (MoCA-MIS) as a predictor of conversion from mild cognitive impairment to Alzheimer's disease. *Journal of the American Geriatrics Society*.

[39] Nazem S., Siderowf A. D., Duda J. E. (2009). Montreal cognitive assessment performance in patients with Parkinson's disease with ‘normal’ global cognition according to mini-mental state examination score. *Journal of the American Geriatrics Society*.

[40] Larner A. J. (2013). Comparing diagnostic accuracy of cognitive screening instruments: a weighted comparison approach. *Dementia and Geriatric Cognitive Disorders Extra*.

[41] Tsai C.-F., Lee W.-J., Wang S.-J., Shia B.-C., Nasreddine Z., Fuh J.-L. (2012). Psychometrics of the Montreal Cognitive Assessment (MoCA) and its subscales: validation of the Taiwanese version of the MoCA and an item response theory analysis. *International Psychogeriatrics*.

[42] Fujiwara Y., Suzuki H., Yasunaga M. (2010). Brief screening tool for mild cognitive impairment in older Japanese: validation of the Japanese version of the Montreal Cognitive Assessment. *Geriatrics and Gerontology International*.

[43] Wong A., Xiong Y. Y., Kwan P. W. L. (2009). The validity, reliability and clinical utility of the Hong Kong Montreal Cognitive Assessment (HK-MoCA) in patients with cerebral small vessel disease. *Dementia and Geriatric Cognitive Disorders*.

[44] Volosin M., Janacsek K., Németh D. (2013). Hungarian version of the Montreal Cognitive Assessment (MoCA) for screening mild cognitive impairment. *Psychiatria Hungarica*.

[45] Costa A. S., Fimm B., Friesen P. (2012). Alternate-form reliability of the montreal cognitive assessment screening test in a clinical setting. *Dementia and Geriatric Cognitive Disorders*.

[46] Costa A. S., Reich A., Fimm B., Ketteler S. T., Schulz J. B., Reetz K. (2014). Evidence of the sensitivity of the MoCA alternate forms in monitoring cognitive change in early alzheimer's disease. *Dementia and Geriatric Cognitive Disorders*.

[47] Litvan I., Bhatia K. P., Burn D. J. (2003). SIC task force appraisal of clinical diagnostic criteria for parkinsonian disorders. *Movement Disorders*.

[48] Zahodne L. B., Young S., Kirsch-Darrow L. (2009). Examination of the Lille Apathy Rating Scale in Parkinson disease. *Movement Disorders*.

[49] Montgomery S. A., Asberg M. (1979). A new depression scale designed to be sensitive to change. *British Journal of Psychiatry*.

[50] Stacho L., Dudás R., Ivady R., Kothencz G., Janka Z. (2003). Addenbrooke's cognitive examination: developing the hungarian version. *Psychiatria Hungarica*.

[51] Hoehn M. M., Yahr M. D. (1967). Parkinsonism: onset, progression and mortality. *Neurology*.

[52] Horváth K., Aschermann Z., Ács P. (2014). Validation of the Hungarian MDS-UPDRS: why do we need a new Parkinson scale?. *Ideggyogyaszati Szemle*.

[53] Horváth K., Aschermann Z., Ács P. (2014). Is the MDS-UPDRS a good screening tool for detecting sleep problems and daytime sleepiness in Parkinson's disease?. *Parkinson's Disease*.

[54] Core Team R. (2015). *R: A Language and Environment for Statistical Computing*.

[55] Robin X., Turck N., Hainard A. (2011). pROC: an open-source package for R and S+ to analyze and compare ROC curves. *BMC Bioinformatics*.

[56] Anang J. B., Gagnon J. F., Bertrand J. A. (2014). Predictors of dementia in Parkinson disease: a prospective cohort study. *Neurology*.

[57] Villeneuve S., Rodrigues-Brazète J., Joncas S., Postuma R. B., Latreille V., Gagnon J. F. (2011). Validity of the Mattis Dementia rating scale to detect mild cognitive impairment in Parkinson's disease and REM sleep behavior disorder. *Dementia and Geriatric Cognitive Disorders*.

[58] Pirogovsky E., Schiehser D. M., Litvan I. (2014). The utility of the Mattis Dementia Rating Scale in Parkinson's disease mild cognitive impairment. *Parkinsonism and Related Disorders*.

[59] van Steenoven I., Aarsland D., Hurtig H. (2014). Conversion between mini-mental state examination, montreal cognitive assessment, and dementia rating scale-2 scores in Parkinson's disease. *Movement Disorders*.

[60] Chou K. L., Lenhart A., Koeppe R. A., Bohnen N. I. (2014). Abnormal MoCA and normal range MMSE scores in Parkinson disease without dementia: cognitive and neurochemical correlates. *Parkinsonism & Related Disorders*.

[61] Karlawish J., Cary M., Moelter S. T. (2013). Cognitive impairment and PD patients' capacity to consent to research. *Neurology*.

[62] Chen L., Yu C., Fu X. (2013). Using the Montreal Cognitive Assessment Scale to screen for dementia in Chinese patients with Parkinson's disease. *Shanghai Archives of Psychiatry*.

[63] Ohta K., Takahashi K., Gotoh J. (2014). Screening for impaired cognitive domains in a large Parkinson's disease population and its application to the diagnostic procedure for Parkinson's disease dementia. *Dementia and Geriatric Cognitive Disorders Extra*.

[64] Kandiah N., Zhang A., Cenina A. R., Au W. L., Nadkarni N., Tan L. C. (2014). Montreal Cognitive Assessment for the screening and prediction of cognitive decline in early Parkinson's disease. *Parkinsonism & Related Disorders*.

[65] Hu M. T., Szewczyk-Królikowski K., Tomlinson P. (2014). Predictors of cognitive impairment in an early stage Parkinson's disease cohort. *Movement Disorders*.

